# Antibacterial Activity of Hydroethanolic Extracts of *Artemisia annua* L., *Hibiscus sabdariffa* L., and *Paronychia argentea* Lam. Against Some Clinically Relevant Gram-Positive and Gram-Negative Bacteria

**DOI:** 10.3390/antibiotics15030249

**Published:** 2026-02-27

**Authors:** Eileen Lendech-Escobar, Ma. Dolores Castañeda-Antonio, Roberto Portillo-Reyes, Jesús Muñoz-Rojas, Ygnacio Martínez-Laguna, Mohamed Abd El-Salam, Bożena Futoma-Kołoch, José Carlos Mendoza-Hernández

**Affiliations:** 1 Faculty of Chemical Sciences, Meritorious Autonomous University of Puebla, Puebla 72590, Mexico; eileen.lendech@alumno.buap.mx (E.L.-E.); roberto.portillo@correo.buap.mx (R.P.-R.); 2 Institute of Sciences, Meritorious Autonomous University of Puebla, Puebla 72590, Mexico; jesus.munoz@correo.buap.mx (J.M.-R.); ignacio.martinez@correo.buap.mx (Y.M.-L.); 3 Department of Pharmacognosy, Faculty of Pharmacy, Delta University for Science and Technology, International Coastal Road, Gamasa 11152, Egypt; mabdelsalam@tecnocampus.cat; 4 Research Group in Hypoxia, Dietetics, Nutrition, and Kinanthropometry (GIHDiNuC), Department of Health, TecnoCampus School of Health Sciences, Pompeu Fabra University, 08302 Barcelona, Catalonia, Spain; 5 Department of Microbiology, Faculty of Biological Sciences, University of Wrocław, Przybyszewskiego 63–77, 51-148 Wroclaw, Poland; 6 Faculty de Chemical Engineering, Meritorious Autonomous University of Puebla, Puebla 72590, Mexico; josecarlos.mendoza@correo.buap.mx

**Keywords:** alternative antibiotics, antibacterial activity, phytochemicals, medicinal plants, gram-positive, gram-negative bacteria

## Abstract

**Background:** Antimicrobial resistance is one of the major challenges in healthcare, leading to more severe infections, higher mortality, and increased healthcare costs. Therefore, exploring new alternatives, such as plant extracts with antimicrobial properties helps to reduce bacterial resistance. *Artemisia annua* L., *Hibiscus sabdariffa* L., and *Paronychia argentea* Lam. are traditionally used for their biological properties, including antimicrobial activity. However, scientific evidence regarding their antibacterial effects against clinically important bacteria remains limited. **Methods:** Extracts were obtained from the mentioned plants and phytochemically characterized by GC/MS. Phytochemical analysis revealed the presence of fatty acids, phenolic compounds, aliphatic compounds, and terpenoids. Antibacterial activity was evaluated using the diffusion method with a modified Kirby-Bauer technique and the microdilution method employing the massive plate sealing drop approach. **Results:** All extracts exhibited bacterial inhibition, with *H. sabdariffa* L. showing the strongest activity against *E. coli* (256 mg/mL), *K. pneumoniae* (256 mg/mL), *P. aeruginosa* (128 mg/mL), *Salmonella* sp. (128 mg/mL), and *S. aureus* (64 mg/mL). **Conclusions:** Hydroethanolic extracts of *A. annua* L., *H. sabdariffa* L., and *P. argentea* Lam. exhibit antibacterial activity against clinically relevant bacteria and represent promising candidates for future preclinical studies.

## 1. Introduction

Antimicrobial resistance (AMR) is considered a “silent pandemic”. According to the WHO GLASS Report 2025, it is associated with approximately 4.7 million deaths annually, and it is estimated that one in six bacterial infections is resistant to conventional treatments. Together these trends represent a major and escalating global public health threat, compromising the effective prevention and treatment of bacterial infections. The urgent need for new antibacterial strategies is therefore driven by the global spread of multidrug-resistant (MDR) pathogens, the decreasing effectiveness of existing antibiotics, and the limited development of new drugs, which together hinder the effective treatment of both community- and hospital-acquired infections [1].

Resistance can emerge through genetic mechanisms, including spontaneous mutations and horizontal gene transfer, as well as through environmental pressures such as stress conditions, altered pH, and prolonged exposure to sub-inhibitory concentrations of antibiotics. These factors, taken together, contribute to increased infection persistence, mortality rates, and a considerable economic burden on healthcare systems, making this problem a priority to address, as stated by the World Health Organization (WHO) [2,3]. The tested bacteria are representatives of the most clinically relevant pathogens and pose a major public health concern. *Escherichia coli* includes pathogenic strains that may exhibit resistance to β-lactams, fluoroquinolones, and, in some cases, extended-spectrum β-lactamases (ESBLs), complicating the treatment of gastrointestinal and urinary tract infections [4,5]. *Klebsiella pneumoniae* (ESBL production and carbapenem resistance) and *Pseudomonas aeruginosa* (resistance to β-lactam, fluoroquinolones, aminoglycosides, carbapenems) are opportunistic pathogens associated with hospital-acquired infections with high levels of intrinsic and acquired multidrug resistance [6,7]. *Salmonella* species have increasingly developed resistance to ampicillin, chloramphenicol, and fluoroquinolones, raising concerns in the management of foodborne infections [8]. *Shigella* species also show growing resistance to first-line antibiotics, including sulfonamides and fluoroquinolones, particularly in endemic regions [9]. *Staphylococcus aureus*, especially methicillin-resistant strains (MRSA), exhibits resistance to β-lactam antibiotics and often to multiple other drug classes, posing a serious challenge in both community- and hospital-acquired infections [10].

The advantage of using natural product extracts as alternatives lies in the fact that, unlike drugs with defined structures and well-established mechanisms, extracts are complex mixtures of bioactive compounds, such as phenols, phenolic acids, tannins, flavonoids, and terpenes that are difficult for bacteria to recognize. As a result, the compounds can disrupt bacterial metabolism and ultimately prevent their survival.

Plant extracts are rich sources of bioactive molecules with antimicrobial, anti-inflammatory, and antioxidant properties, and they continue to provide valuable scaffolds for drug discovery and antibiotic adjuvant development [11].

Mexico boasts an exceptional ethnobotanical culture, standing out as one of the most diverse in the world thanks to its abundant plant and climatic diversity, dating back to pre-Hispanic times. This provides an excellent setting for the study of phytotherapeutic practices, which has been key to the discovery of bioactive compounds. Currently, various plants are consumed in the central region of Mexico, such as San Buenaventura Nealtican, Puebla for kidney problems, but their functional properties still require scientific validation. Therefore, we will now discuss the study of three important plants in the central region of the country that are consumed for antibiotic purposes, aiming to demonstrate their functionality.

*Artemisia annua* L. (Asteraceae), commonly known as sweet wormwood, is a medicinal plant native to China and widely cultivated across temperate and subtropical regions. The plant is characterized by glandular trichomes that secrete volatile oils rich in bioactive constituents. Phytochemical investigations have identified sesquiterpene lactones, flavonoids, coumarins, terpenoids, and essential oils as its major components [12,13]. Beyond its well-established antimalarial activity, *A. annua* has demonstrated antimicrobial, antioxidant, anti-inflammatory, and cytotoxic properties, supporting its potential as a source of antibacterial agents [14]. *Hibiscus sabdariffa* L. (Malvaceae), commonly referred to as roselle, is an annual herbaceous plant cultivated in tropical and subtropical regions worldwide. The calyces of *H. sabdariffa* are rich in organic acids, anthocyanins, phenolic acids, flavonoids, and volatile compounds [15,16,17,18,19,20]. These bioactive constituents have been associated with antioxidant, anti-inflammatory, and antimicrobial activities. In traditional medicine, roselle is widely used for the management of gastrointestinal disorders, urinary tract infections, and inflammatory conditions, further highlighting its pharmacological relevance [17,18,19,20]. *Paronychia argentea* Lam. (Caryophyllaceae) is a perennial medicinal plant native to North Africa that thrives in arid and saline environments. Phytochemical analyses have revealed the presence of phenolic compounds, flavonoids, tocopherols, terpenoids, and phenolic acids, with ferulic acid identified as a predominant constituent [21,22,23]. In ethnomedicine, *P. argentea* is commonly employed in the treatment of renal and urinary tract disorders, gastrointestinal ailments, and infectious diseases, suggesting potential antibacterial efficacy. Despite growing evidence supporting the functional potential of antioxidant and anti-inflammatory compounds in these medicinal plants, there is insufficient data to support their antimicrobial activity. Furthermore, even though the groups of bioactive compounds are known, it is necessary to identify the specific compounds present that could help address the challenge of using them as an alternative to antimicrobial resistance (AMR). Therefore, the aim of our study was to investigate the antibacterial activity of phytoextracts obtained from *A. annua*, *H. sabdariffa*, and *P. argentea* against clinically relevant bacterial pathogens, and to characterize the phytochemical constituents potentially responsible for the observed antibacterial effects.

## 2. Results

**Figure 1:**
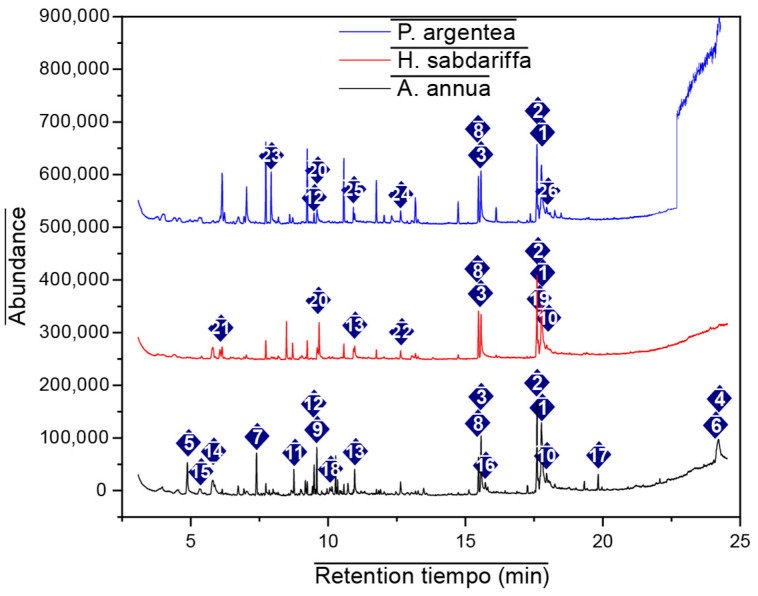
Gas chromatography–mass spectrometry (GC–MS) chromatograms of ethanolic extracts obtained by maceration combined with sonication from *Artemisia annua* (flowers, stems, and leaves), in which 19 bioactive compounds were identified; *Hibiscus sabdariffa* flowers, with 12 detected bioactive compounds; and *Prunus argentea*, with 11 identified constituents. The numbered peaks in the chromatograms correspond to the compounds listed in Table 1.

Gas chromatography coupled with mass spectrometry (GC/MS) was used to identify the chemical constituents and determine their relative abundance in the plant extracts [24,25]. Representative GC/MS chromatograms of the ethanolic extracts of *A. annua*, *H. sabdariffa*, and *P. argentea* are presented in Figure 1.

The secondary metabolites of the ethanolic extract, identified by the NIST08 Library with a quality above 76% of the plants under study, are presented in Table 1, along with their bibliographically reported biological activities.

**Figure 2:**
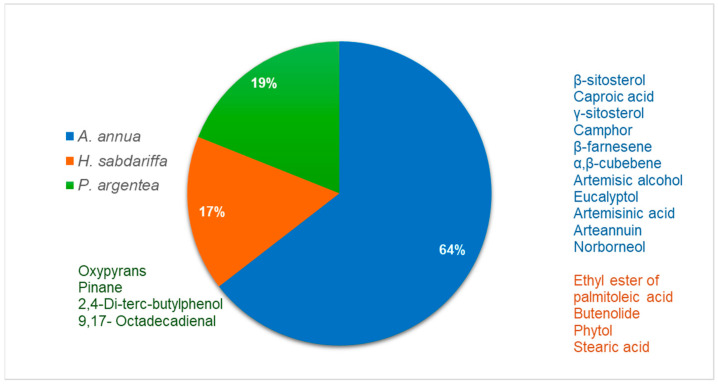
Relative contribution of antimicrobial bioactive compounds identified in each plant species. *A. annua* accounts for 64% of the detected compounds, followed by *H. sabdariffa* 17% and *P. argentea* 19%.

GC/MS analysis confirmed the presence of bioactive compounds reported with anti-inflammatory, anticancer, and hepatoprotective properties, such as phenolic compounds, aliphatic compounds, terpenes, and terpenoids, including monoterpenoids, triterpenoids, and sesquiterpenes. We can observe that although *A. annua* is present at a higher percentage, *H. sabdariffa* exhibits a greater diversity of antimicrobial compounds (Figure 2).

### Antibacterial Activity

Of the six strains tested, inhibition zones were observed in five. Table 2 summarizes the inhibition zone results obtained from evaluating the antibacterial activity of the hydroethanolic extracts against *E. coli*, *K. pneumoniae*, *P. aeruginosa*, *Salmonella* sp., *Shigella* sp., and *S. aureus*. All tests were performed in triplicate.

Table 3 Presents the minimum inhibitory concentrations (MICs) of the hydroethanolic plant extracts against the bacterial strains that showed sensitivity. The MICs were determined using the dilution method with the massive plate sealing drop technique.

**Figure 3:**
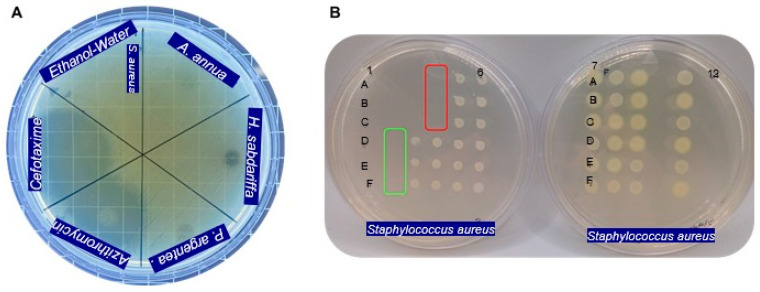
(**A**) Antibacterial activity of the hydroethanolic extracts of *A. annua*, *H. sabdariffa*, and *P. argentea*, evaluated using a modified diffusion assay on Mueller-Hinton agar. Negative control: sterile hydroethanol (50:50, *v*/*v*). Positive controls: azithromycin and cefotaxime (30 mg/mL). (**B**) Minimum inhibitory concentrations (MICs) of *H. sabdariffa* and *P. argentea* extracts determined using the mass-sealing drop method. Red line: MIC of *H. sabdariffa*, 64 mg/mL; green line: MIC of *P. argentea*, 256 mg/mL. All assays were performed in triplicate.

Figure 3 Shows the antibacterial activity of the hydroethanolic extracts against the most susceptible bacterium, *S. aureus*, as evaluated using the Mueller-Hinton agar diffusion assay and the dilution method with the massive plate sealing drop technique.

Due to the color interference of the phytoextracts, we did not use the plate reader to determine the MIC, and the mass plate sealing drop method was carried out using the replicator. This technique allowed us to obtain reliable results and greater precision in the quantifications.

Azithromycin served as a positive control for *E. coli*, *K. pneumoniae*, *Salmonella* sp., *Shigella* sp., and *S. aureus*, while cefotaxime was effective against *P. aeruginosa*. The negative control showed no bacterial inhibition, validating our system. The drugs, as pure compounds, showed the expected efficacy and the bacteria’s sensitivity to them, while the phytocompounds, in their mixture form, demonstrated a synergistic effect on the metabolism of the studied bacteria.

The evaluation of the data obtained by the S-W test ranges from *A. annua* 0.992, *H. sabdariffa* 0.975 and *P. argentea* 0.992 for the normality value, the analysis of variance and Tukey’s test for each bacterium showed a linear correlation in the range of 0.85 to 1.0 for both the extracts and positive controls. The analysis of variance and Tukey’s test for each bacterium showed a significance level of 95% and an α value of 0.05, except for *Shigella* sp., which did not demonstrate inhibitory activity. The *A. annua* extract and the *H. sabdariffa* extract differ significantly in their inhibitory activity with respect to the antibiotic.

## 3. Discussion

The bacteria examined in this study employ a combination of intrinsic and acquired mechanisms that contribute to antimicrobial resistance, including enzymatic antibiotic inactivation (e.g., *K*. *pneumoniae, E*. *coli*), target site modification (e.g., *S*. *aureus*), reduced membrane permeability and active efflux (e.g., *P*. *aeruginosa, Salmonella* spp.). Additional protective strategies such as biofilm formation (e.g., *P*. *aeruginosa, S*. *aureus*) and horizontal gene transfer (e.g., *E*. *coli, Shigella* spp.) further enhance bacterial survival and promote the spread of multidrug resistance. The coexistence of these mechanisms underscores the complexity of antimicrobial resistance and highlights the need for alternative antibacterial approaches capable of overcoming multiple resistance pathways [101,102,103,104,105,106,107,108,109]. Plant-derived extracts, including cranberry polyphenols, cinnamaldehyde, p-coumaric acid, and selected herbal and essential oil extracts, represent a multifaceted strategy against *E. coli*, combining antibacterial, anti-adhesive, antibiofilm and anti-virulence effects. Moreover, by interfering with resistance mechanisms such as efflux pump activity and biofilm formation, these compounds may enhance the efficacy of conventional antibiotics, including fluoroquinolones, aminoglycosides and β-lactams [110].

The resistance of some strains, such as *E. coli*, *K. pneumoniae*, *Shigella* sp., and *P. aeruginosa*, can be attributed to their ability to exhibit efflux to multiple drugs through the action of active efflux pumps. These pumps generate a bacterial defense mechanism against compounds because they have the property of being modified and overexpressed, allowing for a highly efficient defensive process. The drugs are easily detected due to their defined structure; however, the use of extracts containing biocompounds capable of permeating the pumps and accumulating in the cytoplasm, causing toxicity, can be problematic [111].

*A. annua* has been studied as an essential oil, reporting the camphor/artemisia ketone biocompounds through disc impregnation giving inhibition halos tested on *Bacillus subtilis*, *E. coli*, *S. aureus*, *S. typhimurium*, with an MIC of 4% without mentioning the initial concentration, although its inhibition halos are reported similar to that being reported in this work for *Salmonella* sp. of 10.14 mm at a concentration of 512 mg/L, it has also been reported by maceration with ethanol and dimethyl sulfide inhibiting *E. coli* and *S. aureus* at MIC = 62.5 μg/mL and MIC = 125 μg/mL, respectively, although the reported concentrations are lower the use of solvent could have influenced since the controls used are not mentioned [12,112].

The essential oils of *A. annua*, containing artemisia ketone, caryophyllene, and β-selinene, may increase their efficacy against Gram-negative bacteria by acting synergistically with each other, facilitating their entry into the cell membrane due to their lipophilic properties. On the other hand, the aliphatic chain of the essential oil could be accumulating in the cytoplasmic membranes of the bacteria, leading to impaired membrane permeability, causing malfunction, and ultimately, the rapid death of the microorganisms [113].

The compounds with the highest proportion in *H. sabdariffa* were oleic acid (20.29%), ethyl oleate (13.05%), palmitic acid (10.67%), ethyl palmitate (6.80%), and 1,4-dichlorobenzene (4.50%). In contrast, the compounds with the lowest concentration in the extract were phytol (1.73%), stearic acid (1.33%), and linoleic acid (1.12%). Inhibition was obtained from 4.07 mm for *Salmonella* sp. to 6.53 mm for *E. coli*. This extract has been tested on other bacteria, as reported by [114], causing inhibition in *Streptococcus mutans*, *Streptococcus sanguinis*, *Capnocytophaga gingivalis*, and *S. aureus* at MIC: of 5–7 mg/mL, using acetone and ethyl acetate as solvents, which coincides in very similar values but has the advantage of using a less toxic solvent and reporting the compounds present in the hydroethanolic extract, other report using *H. sabdariffa* pigment combined with ascorbic acid, citric acid and malic acid at a concentration lower than 10.26 mg/L, for bacteria *Streptococcus mutans*, *Aggregatibacter actinomycetemcomitans*, *Prevotella intermedia*, however this study alters pH conditions which could also be affecting the inhibition [115].

**Figure 4:**
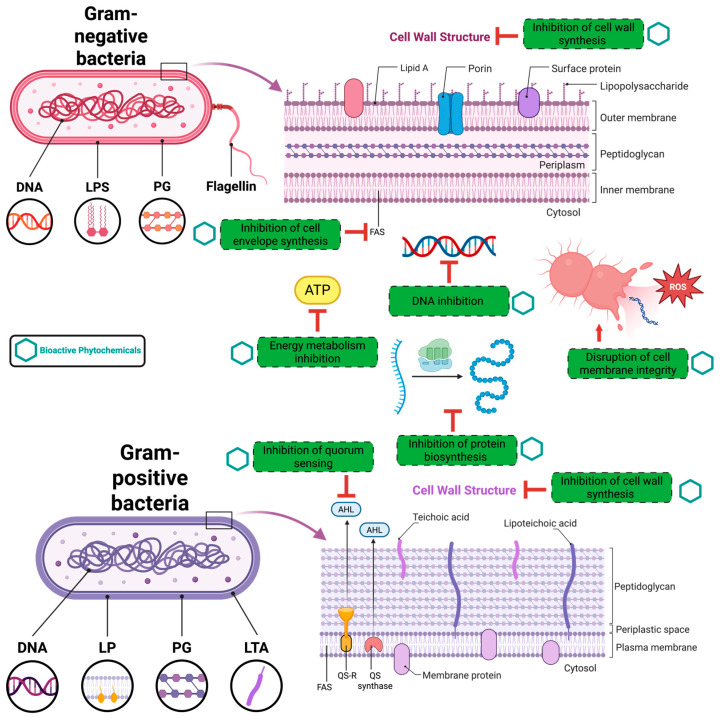
Schematic representation of the principal antibacterial targets of bioactive phytochemicals in Gram-positive and Gram-negative bacteria. Plant-derived compounds can act on multiple cellular systems simultaneously. In QS inhibition, phytochemicals may block the production or perception of signaling molecules such as acyl-homoserine lactones (AHLs) or interfere with their binding to the quorum sensing regulator/receptor (QS-R), thereby reducing the expression of genes involved in virulence, biofilm formation, and resistance. The multitarget nature of phytochemicals highlights their therapeutic potential as antimicrobial and anti-virulence agents and supports their relevance in strategies aimed at combating antimicrobial resistance. Adapted and modified from [116].

The reported mechanisms of action involve multiple bacterial targets. These include disruption of membrane integrity and increased permeability, effects commonly asso-ciated with essential oils, terpenes, flavonoids, and saponins. Impairment of cellular energy metabolism has been attributed to polyphenols and camphor, while the induction of oxidative stress is primarily linked to polyphenolic constituents. In addition, flavonoids and fatty acids have been shown to interfere with nucleic acid synthesis, affecting both DNA replication and RNA transcription. Inhibition of protein biosynthesis has likewise been related to the activity of flavonoids, polyphenols, and fatty acids. Artemisinin has been reported to reduce efflux pump activity, thereby potentially enhancing intracellular drug accumulation. Polyphenols also play an important role in suppressing bacterial biofilm formation. Further proposed mechanisms include interference with cell envelope assembly and inhibition of cell wall biosynthesis, actions mainly attributed to polyphenols, flavonoids, and fatty acids (Figure 4) [116].

*P. argentea* inhibited *E. coli* at >512 mg/mL, *K. pneumoniae* at 512 mg/mL, and *S. aureus* at 256 mg/mL. Saponins present in the extract likely contribute to its greater efficacy against Gram-positive bacteria by binding to membrane sterols, disrupting the cell wall and cytoplasmic membrane, and causing leakage of cellular contents [50].

Strong antibacterial activity against *Shigella sonnei* and *Shigella flexneri* has been reported at concentrations tested 3.125–100 mg/mL using aqueous phytoextracts of *T. brownii* [117]. However, our study showed that the *Shigella* sp. used in this study was resistant to the three extracts, suggesting that it is resistant since the positive controls also did not cause an inhibitory response due to its complex Gram-negative cell envelope, low permeability to phytocompounds, active efflux pumps, or enzymatic hydrolysis of bioactive compounds [118].

## 4. Materials and Methods

### 4.1. Plant Material

Dried *H. sabdariffa* L. flowers were obtained from Tzicatlán, Puebla, Mexico (GMS N 18° 27′ 15.001″ O 98° 45′ 20.001″), a municipality where this plant is cultivated. Flowers, stems, and leaves of *A. annua* L., were collected from the Botanical Garden of Tlaxcala, Mexico (GMS N 19° 19′ 35.796″ O 98° 22′ 38.855″), and *P. argentea* Lam. leaves and roots were obtained from the community of San Buenaventura Nealtican, Puebla, Mexico (GMS N 18° 27′ 15.001″ W 98° 45′ 20.001″). Specimens were collected in sufficient quantities for the proposed trials in October 2024. All plant material was air-dried before extraction. All plants were authenticated and identified using the “Picture This” application and deposited at the Institute of Sciences of the Benemérita Universidad Autónoma de Puebla, Mexico.

### 4.2. Phytochemical Characterization

The phytoextracts used for characterization were prepared using ethanol, analytical reagent grade, by maceration at room temperature for 24 h with occasional stirring. The resulting concentration was 2:1, yielding a dry extract, corresponding to 2 g of dry plant material per 1 mL of solvent. This was followed by ultrasound-assisted extraction (Sunne brand ultrasonic bath) at a frequency of 40 kW at 25 °C for 10 min. The extracts were filtered and stored under refrigeration at 4 °C until subsequent chromatographic analysis, solvent blanks were run before each sample, compound identification was performed using the NIST08 library, and each injection was performed in triplicate.

The extracts were analyzed using an Agilent Technologies 7890A gas chromatograph coupled to an Agilent 5975C mass spectrometer (Agilent Technologies, Santa Clara, CA, USA). Separation was achieved on a Zebron ZB-50 column (30 m × 0.25 mm i.d., 0.25 μm film thickness) (Phenomenex, Torrance, CA, USA). The oven temperature program was set as follows: 60 °C for 1 min, ramped at 12 °C/min to 194 °C and held for 2 min, then increased at 10 °C/min to 295 °C and held for 4 min. The injector temperature was 250 °C, with helium as the carrier gas at a flow rate of 1.3 mL/min. Masses of 35 to 500 amu were detected, the fragmentation is carried out by electronic impact at 70 eV, one microliter of each sample was injected in splitless mode, and compound identification was performed using the NIST 08 mass spectrometry library [119].

### 4.3. Preparation of Hydroethanolic Extracts for Inhibition Tests

Plant extracts for inhibition tests were prepared using ethanol (96% GRA) and isotonic water in a 1:1 ratio (ethanol:water). Maceration was performed for 24 h at room temperature, followed by ultrasound-assisted extraction (brand Luzeren, mod LUZ-21052, 360 W) at 25 °C for 10 min. The drug-to-extract ratio (DER) was 2:1, corresponding to 2 g of dried plant material per 1 mL of hydroethanolic solvent. The extracts were then filtered through 11 µL Whatman filter paper to remove particles, followed by sterilization by filtration with an MCE membrane of 0.22 µm (Millipore Sigma (Merck KGaA), Burlington MA, USA). All extracts were stored at 4 °C until further analysis [25,119,120].

### 4.4. Bacterial Strains and Culture Conditions

Antibacterial activity was evaluated against six clinically relevant bacterial strains: *Escherichia coli*, *Klebsiella pneumoniae*, *Pseudomonas aeruginosa*, *Salmonella* spp., *Shigella* sp., and *Staphylococcus aureus*. All strains correspond to bacteria clinically isolated from patients without reported antimicrobial resistance, characterized and maintained in the laboratory and collected by the culture collection of the Faculty of Chemical Sciences, Benemérita Universidad Autónoma de Puebla (BUAP).

Prior to testing, bacterial identity was confirmed according to Cowan and Steel criteria, including Gram staining, metabolic profiling, catalase and oxidase activity, motility, carbohydrate fermentation, indole production, citrate utilization, and glucose acidification. Strains were cultured on Trypticase Soy Agar (TSA), (Becton Dickinson (BD) Cuautitlán Izcalli, México) and, where appropriate, on selective and differential media, including MacConkey agar (MC) (brand BIOXON, MCD, LAB S.A. de C.V., Tlalnepantla, México) and Mannitol Salt Agar (MSA) (brand BIOXON, MCD, LAB S.A. de C.V., Tlalnepantla, México) [121].

Bacterial colonies grown on nutrient agar (AN) (brand BIOXON, MCD, LAB S.A. de C.V., Tlalnepantla, México) were incubated at 36 ± 1 °C for 16–18 h. Bacterial suspensions were adjusted using a McFarland calibration curve to achieve the desired inoculum density. Post-culture purity was verified by visual inspection of colony morphology and subculturing on non-selective agar. All procedures were performed in accordance with CLSI/EUCAST guidelines [121,122].

### 4.5. Agar Diffusion Assay

Antibacterial activity was initially assessed using the agar diffusion method based on a modified Kirby–Bauer technique, where instead of impregnated discs, a direct drop was applied to the agar. Mueller–Hinton agar (brand BIOXON, MCD, LAB S.A. de C.V., Tlalnepantla, México) was inoculated with standardized bacterial suspensions by mass plating, and 4 µL drops of each positive control (azithromycin and cefotaxime at a concentration of 30 mg/mL), negative control (sterile hydroethanol (50:50) and phytoextract were applied to the agar surface. The plates were incubated at 37 °C, after which the zones of inhibition were measured, the inhibition zones were measured. The criterion for determining the sensitivity of the bacteria to the phytoextracts was based on the droplet diameter of the phytoextracts and antibiotics; >4.0 mm was classified as sensitive bacteria and <4.0 mm as resistant bacteria to the phytoextract or antibiotic. All experiments were performed in triplicate [119,120,121,122,123].

### 4.6. Determination of Minimum Inhibitory Concentration (MIC)

**Figure 5:**
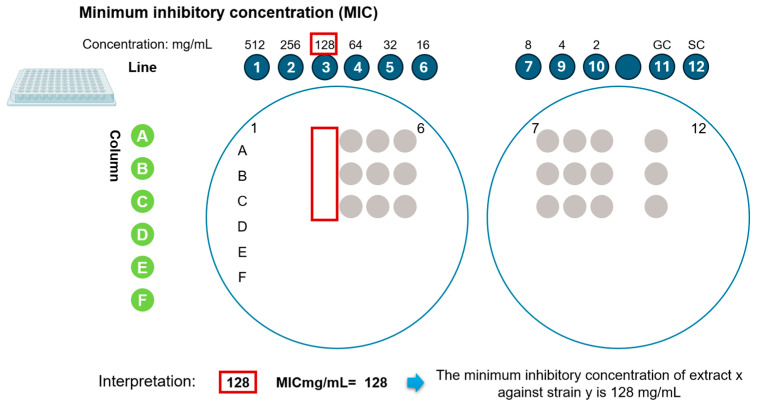
Interpretation of inhibition in the microdilution assay combined with the sealed-plate method (SPM). GC (−), growth control; SC (+), sterility control.

The minimum inhibitory concentration (MIC) was determined using a microdilution assay in 300 µL plates, in combination with Massive Stamping Drop (MSDP) methodology [109]. Mueller–Hinton broth (BD Bioxon) was used as the culture medium. Phytoextracts were initially prepared at a concentration of 512 mg/mL and serially diluted to obtain concentrations ranging from 512 to 2 mg/mL. Following incubation at 37 °C for 18 h under shaking conditions, aliquots were transferred to Mueller–Hinton agar plates using the drip-by-mass method. Plates were incubated for an additional 18–24 h, and the MIC was defined as the lowest concentration of extract that completely inhibited visible bacterial growth (Figure 5) [121,124].

### 4.7. Statistical Analysis

The normality of the data was determined by the Shapiro-Wilk test and homogeneity of variances with the Levene test to carry out an analysis of variance (ANOVA) with a significance level of *p* ≤ 0.05. Tukey’s post hoc test was applied to identify statistically significant differences among treatments at a 95% confidence interval. Dates were analyzed using Minitab Statistical Software version 22.

## 5. Conclusions

Resistance to conventional antibiotics remains a serious global health problem, making the search for natural products with antimicrobial activity a promising approach to discovering new molecules effective against antibiotic-resistant bacteria, thus expanding therapeutic options. This in vitro study demonstrated the antibacterial activity of hydroethanolic extracts of *A. annua*, *H. sabdariffa*, and *P. argentea* against clinically virulent bacteria. The *H. sabdariffa* extract was the most effective against *S. aureus*, while the other extracts showed moderate antibacterial activity against other tested strains. Specific compounds were detected by mass spectrometry identification, which will allow for their isolation and quantification in the future. An alternative technique, the mass sealing drip method, is also proposed. This method provides accurate evaluation when the color of the extracts interferes with quantification by plate reader.

## Figures and Tables

**Table 1:** Compounds identified by GC/MS in the ethanolic extracts of *Artemisia annua*, *Hibiscus sabdariffa*, and *Prunus argentea*.

Phytochemical Compound		Plant Where It Was Found (%)	Reported Activities	Reference
**1**	Oleic acid 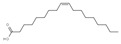	*A. annua* (20.20) *H. sabdariffa* (20.29) *P. argentea* (11.77)	Antioxidant, anti-inflammatory, antitumor, immunostimulant, antiandrogenic, antibacterial, antifungal, acts as a lubricant, binder, anti-foaming agent, important factor in the hypoglycemic effect, pharmaceutical solvent	[26,27,28,29]
**2**	Ethyl oleate 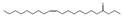	*A. annua* (7.6) *H. sabdariffa* (13.05) *P. argentea* (10.06)	Acaricide, solvent in pesticide products	[30,31]
**3**	Palmitic acid 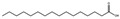	*A. annua* (6.19) *H. sabdariffa* (10.67) *P. argentea* (7.16)	Lubricant, binder, antifoaming agent, disinfectant, antioxidant, anti-inflammatory, hypocholesterolemic, antiandrogenic, antibacterial, antifungal	[29,31,32,33,34]
**4**	β-sitosterol 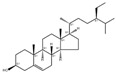	*A. annua* (6.04)	Antioxidant, anticancer, anti-inflammatory, angiogenic, chemopreventive, immunomodulatory and antilipemic	[35,36]
**5**	Caproic acid 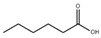	*A. annua* (3.97)	Effective against bacterial and fungal plant pathogens without causing phytotoxicity in crops	[37,38,39]
**6**	γ-sitosterol 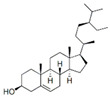	*A. annua* (3.63)	Anticancer	[40,41]
**7**	Camphor 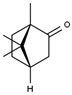	*A. annua* (3.12)	Cutaneous antipruritic, anti-infective and antimicrobial	[42,43,44]
**8**	Ethyl palmitate 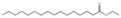	*A. annua* (2.72) *H. sabdariffa* (6.80) *P. argéntea* (4.26)	Anti-inflammatory, antimicrobial, antioxidant, hypocholesterolemic, nematicide, flavoring agent, lubricant, emollient in cosmetics	[45,46,47]
**9**	β-farnesene 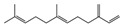	*A. annua* (2.44)	Repellent against pest aphid species	[48,49]
**10**	Linoleic acid 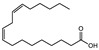	*A. annua* (2.18) *H. sabdariffa* (2.45)	Anti-inflammatory, antibacterial, moisturizing, healing, improves the effectiveness of anticancer drugs, prevents hyperlipidemia, reduces the risk of cardiovascular diseases, treats dietary deficiency or imbalance, emulsifier, cleanser, emollient and skin conditioner	[50,51,52,53]
**11**	α,β-cubebene 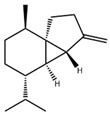	*A. annua* (3.77)	Neuroprotective effects, Antioxidant and antibacterial properties. Plant metabolite, antibacterial and antifungal	[54,55,56,57,58,59,60]
**12**	Caryophyllene 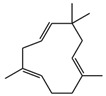	*A. annua* (1.69) *P. argentea* (0.84)	Non-steroidal anti-inflammatory drug, fragrance, metabolite, insect attractant, antioxidant, antifungal, antibacterial, and with cytotoxic properties	[61,62,63]
**13**	Lauric acid 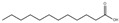	*A. annua* (1.58) *H. sabdariffa* (3.12)	Bactericidal properties, antibacterial agent, protect against Alzheimer’s disease, lubricant, binder and antifoaming agent	[64,65]
**14**	Artemisia alcohol 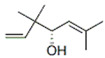	*A. annua* (1.49)	Antioxidant, antibacterial and antifungal	[66,67,68]
**15**	Eucalyptol 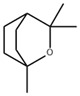	*A. annua* (1.20)	Air freshener, fragrance, disinfectant, solvent, treatment for rhinosinusitis, control of mucus hypersecretion and asthma	[69,70]
**16**	Artemisinic acid 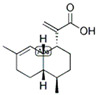	*A. annua* (1.16)	Antimalarial, antitumor, antipyretic, antibacterial, allelopathic, and antiadipogenic	[71,72]
**17**	Arteannuin B 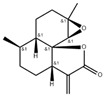	*A. annua* (1.07)	Anti-inflammatory, antiviral (coronavirus), antibacterial, anticancer, pro-apoptotic, antipyretic, antioxidant and immunomodulatory	[73,74,75,76,77,78]
**18**	Norborneol 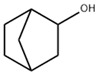	*A. annua* (0.95)	Antimicrobial, insecticide	[79,80,81,82]
**19**	Ethyl ester of palmitoleic acid 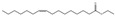	*H. sabdariffa* (2.62)	Effective against pancreatitis and has an antimicrobial effect	[83,84,85]
**20**	5-hydroxymethyl-2-furaldehyde 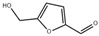	*H. sabdariffa* (2.44) *P. argentea* (2.33)	Quality indicator in food products, antioxidant, suppresses the production of quorum sensing (QS) controlled virulence phenotypes and biofilm formation in *P. aeruginosa*, used in the synthesis of prepolymer and antiviral precursor, 5,5’oxy(bismethylene)-2-furaldehyde (OBMF)	[86,87,88]
**21**	Butenolide 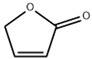	*H. sabdariffa* (2.05)	Flavoring agent, appetite suppressant, immunosuppressant, analgesic, anti-inflammatory, anticancer, anticonvulsant, antimi’crobial, antioxidant, antiulcer, and antituberculosis agent. Inhibits quorum sensing-mediated behaviors.	[89,90,91,92]
**22**	Phytol 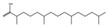	*H. sabdariffa* (1.12)	Anxiolytic, cytotoxic, metabolism modulator, antioxidant, autophagy and apoptosis inducer, anti-nociceptive, anti-inflammatory, immunomodulatory, antibacterial, antifungal, antimalarial, analgesic, and anticarcinogenic effects	[93,94,95]
**23**	Stearic acid 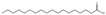	*H. sabdariffa* (1.33)	Antibacterial, antifungal, lubricant, binder, antifoaming agent, pharmaceutical adjuvant	[96,97,98]
**24**	Oxypyrans, pyranones 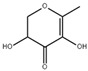	*P. argentea* (5.28)	Antioxidant, antibacterial and antifungal, breaks DNA chains	[99,100,101,102]
**25**	(-)-trans-Pinane 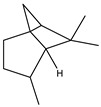	*P. argentea* (1.38)	Aromatic and flavoring agent, insect repellent, antimicrobial	[103,104]
**26**	2,4-Di-terc-butilfenol 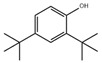	*P. argentea* (1.26)	Bacterial metabolite, antioxidant, and marine metabolite	[105,106]
**27**	9,17- Octadecadienal, (Z) 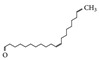	*P. argentea* (0.94)	Insecticide, anti-eczematic, nematicide, antihistamine and antimicrobial	[107,108]

**Table 2:** Antibacterial activity of ethanolic extracts from *Artemisia annua*, *Hibiscus sabdariffa*, and *Prunus argentea* against selected bacterial strains.

Inhibition Zone (mm)						
Bacteria	*A. annua* F-T/H	*H. sabdariffa* F	*P. argentea* H/R	Azithromycin	Cefotaxime	*p*-Value
*E. coli*	NA	6.53 ± 2.40 ^A^	7.36 ± 2.03 ^A^	18.53 ± 0.52 ^B^	32.40 ± 1.75	0.0003462
*K. pneumoniae*	NA	5.66 ± 1.75 ^A^	6.04 ± 1.25 ^A^	8.33 ± 0.39 ^A^	15.98 ± 0.82	0.0821665
*P. aeruginosa*	NA	5.59 ± 0.39 ^A^	NA	NA	19.23 ± 1.76 ^B^	0.0001955
*Salmonella* sp.	10.14 ± 0.98 ^A^	4.07 ± 0.03 ^B^	NA	20.73 ± 0.92 ^C^	31.06 ± 0.94	0.0000006
*Shigella* sp.	NA	NA	NA	21.00 ± 0.14	33.88 ± 0.65	ND
*S. aureus*	NA	5.45 ± 1.10 ^A^	6.34 ± 1.19 ^A^	22.97 ± 0.54 ^B^	28.61 ± 0.67	0.0000009

*A. annua* F–T/H: flower–stem/leaf; *H. sabdariffa* F: flower; *P. argentea* H/R: leaf/root; NA: no activity; ND: not detectable. Note: Means with different superscript capital letters within the same row indicate significant differences (*p* < 0.05), while means with the same superscript capital letters indicate no significant difference (*p* > 0.05).

**Table 3:** Minimum inhibitory concentrations (MICs) of hydroethanolic plant extracts against clinically relevant bacterial strains.

Minimum Inhibitory Concentration (MIC) in mg/mL			
Bacteria	Hydroethanolic Extracts		
	*A. annua*	*H. sabdariffa*	*P. argentea*
*E. coli*	ND	256	>512
*K. pneumoniae*	ND	256	512
*P. aeruginosa*	ND	128	ND
*Salmonella* sp.	512	128	ND
*S. aureus*	ND	64	256

ND: not detectable.

## Data Availability

Available upon request from the main corresponding author.
